# Toward Sustainable Environmental Quality: Priority Research Questions for Asia

**DOI:** 10.1002/etc.4788

**Published:** 2020-07-20

**Authors:** Kenneth M.Y. Leung, Katie W.Y. Yeung, Jing You, Kyungho Choi, Xiaowei Zhang, Ross Smith, Guang‐Jie Zhou, Mana M.N. Yung, Carlos Arias‐Barreiro, Youn‐Joo An, S. Rebekah Burket, Robert Dwyer, Nathalie Goodkin, Yii Siang Hii, Tham Hoang, Chris Humphrey, Chuleemas Boonthai Iwai, Seung‐Woo Jeong, Guillaume Juhel, Ali Karami, Katerina Kyriazi‐Huber, Kuan‐Chun Lee, Bin‐Le Lin, Ben Lu, Patrick Martin, Mae Grace Nillos, Katharina Oginawati, I.V.N. Rathnayake, Yenny Risjani, Mohammad Shoeb, Chin Hon Tan, Maria Claret Tsuchiya, Gerald T. Ankley, Alistair B.A. Boxall, Murray A. Rudd, Bryan W. Brooks

**Affiliations:** ^1^ Swire Institute of Marine Science and School of Biological Sciences University of Hong Kong Pokfulam Hong Kong China; ^2^ State Key Laboratory of Marine Pollution and Department of Chemistry City University of Hong Kong Kowloon Hong Kong China; ^3^ School of Environment and Guangdong Key Laboratory of Environmental Pollution and Health Jinan University Guangzhou China; ^4^ Seoul National University Seoul Korea; ^5^ School of the Environment Nanjing University Nanjing China; ^6^ Hydrobiology Brisbane Queensland Australia; ^7^ Open University of Hong Kong Hong Kong China; ^8^ PETRONAS Kuala Lumpur Malaysia; ^9^ Konkuk University Seoul Korea; ^10^ Baylor University Waco Texas USA; ^11^ International Copper Association Washington DC USA; ^12^ Nanyang Technological University Singapore; ^13^ University of Malaysia Terengganu Malaysia; ^14^ Loyola University Chicago Illinois USA; ^15^ Supervising Scientist Branch Canberra Australian Capital Territory Australia; ^16^ Khon Kaen University Khon Kaen Thailand; ^17^ Kunsan National University Gunsan Korea; ^18^ National University of Singapore Singapore; ^19^ Universiti Putra Serdang Malaysia; ^20^ Corteva Agriscience Geneva Switzerland; ^21^ Proctor and Gamble Singapore; ^22^ National Institute of Advanced Industrial Science and Technology Tokyo Japan; ^23^ International Copper Association–Asia Shanghai China; ^24^ College of Fisheries and Ocean Sciences University of the Philippines Visayas Iloilo City Philippines; ^25^ Bandung Institute of Technology Bandung Indonesia; ^26^ Department of Microbiology Faculty of Science, University of Kelaniya Kelaniya Sri Lanka; ^27^ Universitas Brawijaya Malan Indonesia; ^28^ University of Dhaka Dhaka Bangladesh; ^29^ University of the Philippines Los Baños Los Baños Philippines; ^30^ US Environmental Protection Agency Washington DC; ^31^ University of York York United Kingdom; ^32^ World Maritime University Malmo Sweden

**Keywords:** Environmental chemistry, Environmental toxicology, Hazard/risk assessment, Biomonitoring, Climate change

## Abstract

Environmental and human health challenges are pronounced in Asia, an exceptionally diverse and complex region where influences of global megatrends are extensive and numerous stresses to environmental quality exist. Identifying priorities necessary to engage grand challenges can be facilitated through horizon scanning exercises, and to this end we identified and examined 23 priority research questions needed to advance toward more sustainable environmental quality in Asia, as part of the Global Horizon Scanning Project. Advances in environmental toxicology, environmental chemistry, biological monitoring, and risk‐assessment methodologies are necessary to address the adverse impacts of environmental stressors on ecosystem services and biodiversity, with Asia being home to numerous biodiversity hotspots. Intersections of the food–energy–water nexus are profound in Asia; innovative and aggressive technologies are necessary to provide clean water, ensure food safety, and stimulate energy efficiency, while improving ecological integrity and addressing legacy and emerging threats to public health and the environment, particularly with increased aquaculture production. Asia is the largest chemical‐producing continent globally. Accordingly, sustainable and green chemistry and engineering present decided opportunities to stimulate innovation and realize a number of the United Nations Sustainable Development Goals. Engaging the priority research questions identified herein will require transdisciplinary coordination through existing and nontraditional partnerships within and among countries and sectors. Answering these questions will not be easy but is necessary to achieve more sustainable environmental quality in Asia. *Environ Toxicol Chem* 2020;39:1485–1505. © 2020 The Authors. *Environmental Toxicology and Chemistry* published by Wiley Periodicals LLC on behalf of SETAC.

## INTRODUCTION

**Figure 1:**
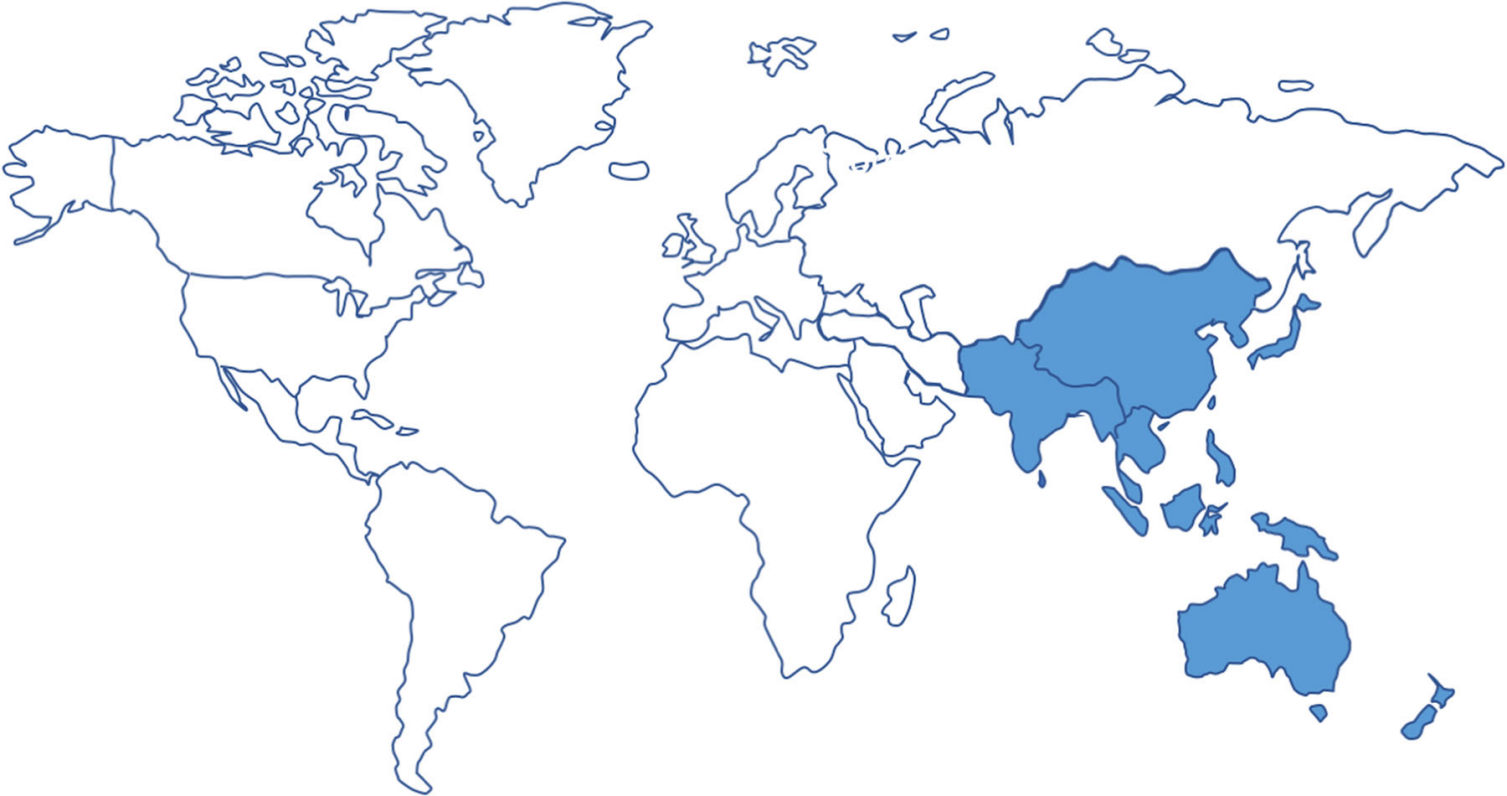
Area coverage of Asia‐Pacific region (modified from United Nations Regional Commissions New York Office 2017). It is important to note that SETAC Asia Pacific Geographic Unit does not include members from Georgia, Middle East, Russia, and Turkey.

Environmental and human health challenges in Asia are pronounced and increasingly influenced by global megatrends spanning rural to urban and industrial gradients and interactions within and among countries, which vary across all stages of development. Located within the broader Asia Pacific region, which extends from the western Pacific Ocean to the Russian Federation in the north, New Zealand in the south, Turkey in the west, to Kiribati in the east (shaded area in Figure 1; United Nations Regional Commissions New York Office 2017), Asia is the largest continent in the world, covering 29.4% of the Earth's land surface (International Maritime Organization 2017). In contrast, Oceania is the smallest continent, consisting of thousands of islands (*National Geographic* 2012). The Asia Pacific region is home to approximately 4.1 billion people, making up more than half of the world's population of approximately 7.5 billion in 2017 (Table 1; International Maritime Organization 2017; Population Reference Bureau 2017; United Nations Economic and Social Commission for Asia and the Pacific 2017). Of the top 10 most populated countries, 6 (i.e., China, India, Indonesia, Pakistan, Bangladesh, and Russia) are within Asia (Population Reference Bureau 2017). By 2030, Asia will include 22 megacities, and it is urbanizing, along with Africa, faster than other continents (United Nations 2014). With such population growth and urbanization come challenges for waste management, and Asia has been predicted to become the leading generator of global municipal solid waste by 2030 (United Nations Environment Programme 2015).

**Table 1:** Summary of the total population, income classification, and per capita GDP of Asia‐Pacific countries and areas listed in United Nations (World Bank 2016, 2017b; Population Reference Bureau 2017)

Country/area	Total population (in millions; mid‐2017)	Population density (per square kilometer of arable land; in thousands)	Income classification^a^ (June 2017)	Per capita GDP (in current USD; 2016)	Sewage treatment facilities
East and Northeast Asia					
Mainland China	1386.8	1312	Upper middle	8109	Mostly equipped with biological treatment facilities^b^
Democratic People's Republic of Korea	25.5	1085	Low	648	NA
Republic of Korea	51.4	3482	High	27 397	Biological treatment^b^
Hong Kong SAR	7.4	238 710	High	42 431	Biological treatment
Macao SAR	0.6	NA	High	78 586	Biological treatment
Japan	126.7	3000	High	34 629	Biological treatment^b^
Mongolia	3.2	564	Lower middle	3973	Biological treatment^b^
Southeast Asia					
Brunei Darussalam^c^	0.4	8000	High	30 553	Biological treatment
Cambodia^c^	15.9	418	Lower middle	1159	Biological treatment for industrial wastewater only; others are directly discharged to rivers
Indonesia^c^	264.0	1123	Lower middle	3346	Septic tanks^b^
Lao People's Democratic Republic^c^	7.0	459	Lower middle	1850	Septic tanks^b^
Malaysia^c^	31.6	3312	Upper middle	9768	Biological treatment covering 70% of the population only^b^
Myanmar^c^	53.4	495	Lower middle	1161	20% Biological treatment; 80% septic tanks^b^
Philippines^c^	105.0	1878	Lower middle	2904	15% biological treatment; 85% septic tanks^b^
Singapore^c^	5.7	1 017 857	High	52 239	Biological treatment
Thailand^c^	66.1	393	Upper middle	5815	Biological treatment for part of the population^b^
Timor‐Leste^c^	1.3	839	Lower middle	2425	Septic tanks^b^
Vietnam^c^	93.7	1462	Lower middle	2068	Only 20% wastewater is treated; others are directly discharged to rivers
South and Southwest Asia					
Afghanistan	35.5	457	Low	623	Septic tanks^b^
Bangladesh^c^	164.7	2148	Lower middle	1208	Septic tanks but not functioning well^b^
Bhutan^c^	0.8	798	Lower middle	2677	Upgrading to biological treatment^b^
India^c^	1352.6	865	Lower middle	1614	Biological treatment but only 40% wastewater is treated^b^
Maldives	0.4	10 256	Upper middle	9446	Septic tanks^b^
Nepal^c^	29.4	1391	Low	725	Sewage treatment plants not working
Pakistan^c^	199.3	655	Lower middle	1410	Chemical‐enhanced primary sedimentation^b^
Sri Lanka^c^	21.4	1646	Lower middle	3974	Biological treatment for industrial wastewater; septic tanks for domestic wastewater^b^
North and Central Asia					
Armenia	3.0	670	Lower middle	3489	Septic tanks and latrines
Azerbaijan	9.9	514	Upper middle	5439	Septic tanks
Kazakhstan	18.0	61	Upper middle	10 312	Biological treatment for urban area; septic tanks for rural area
Kyrgyzstan	6.2	484	Lower middle	1106	Septic tanks^b^
Tajikistan	8.8	1206	Lower middle	926	Biological treatment but not fully utilized^b^
Turkmenistan	5.8	299	Upper middle	6997	Biological treatment^b^
Uzbekistan	32.4	736	Lower middle	2308	Septic tanks
Oceania					
American Samoa	0.06	NA	Upper middle	NA	Primary treatment^b^
Australia	24.5	52	High	51 352	Biological treatment; majority using septic tanks^b^
Cook Islands	0.02	NA	NA	14 119	Septic tanks
Fiji	0.9	546	Upper middle	4922	Biological treatment
French Polynesia	0.3	12 000	High	18 161	Septic tanks
Guam	0.2	20 000	High	NA	Secondary treatment
Kiribati^c^	0.1	5000	Lower middle	1443	Septic tanks^b^
Marshall Islands^c^	0.06	3000	Upper middle	3453	Septic tanks but not functioning well
Federated States of Micronesia^c^	0.1	5000	Lower middle	3015	Septic tanks but not functioning well
Nauru	0.01	NA	Upper middle	18 469	Septic tanks and pit latrine
New Caledonia	0.3	4808	High	33 966	Not having a well‐organized sewage system
New Zealand	4.8	814	High	38 294	Biological treatment^b^
Niue	0.002	NA	NA	NA	Septic tanks
Northern Mariana Islands	0.06	NA	High	NA	Septic tanks
Palau	0.02	2000	High	12 122	Building advanced treatment facilities
Papua New Guinea^c^	8.3	2767	Lower middle	2798	Septic tanks; upgrading to biological treatment by 2018^b^
Samoa	0.2	2500	Upper middle	4006	Septic tanks and pit latrine^b^
Solomon Islands	0.7	3500	Lower middle	1842	Septic tanks^b^
Tonga	0.1	556	Upper middle	3784	Septic tanks and pit latrine^b^
Tuvalu	0.01	NA	Upper middle	3362	Pit latrine
Vanuatu	0.3	1500	Lower middle	2783	Septic tanks for urban area; pit latrines for rural areas^b^

It is noted that SETAC Asia Pacific Geographic Unit does not include members from Georgia, Middle East, Russia and Turkey.

^a^ Income group was classified into 4 categories: low (<$1005), lower middle ($1006–3955), upper middle ($3956–12 235), and high (>$12 236).

^b^ Indicates that information was obtained from the capital only.

^c^ Indicates countries/areas that lie in the tropics.

GDP = gross domestic product; NA = no data available; SAR = Special Administrative Region; USD = US dollars.

Though it is difficult to single out a representative primary industry in Asia because of varying development levels among countries, which gives rise to very diverse and complex industry types, countries tend to shift from agriculture to manufacturing and then eventually to services (Economy Watch 2010). In more developed and high‐income areas, such as Hong Kong, Singapore, and Japan, gross domestic products (GDPs) are primarily generated by providing services because the agriculture and manufacturing industries declined some decades ago (Economy Watch 2010). For emerging countries like China, industries are now shifting to provide services with a lesser focus on the manufacturing industry. Less developed countries, such as Vietnam and India, focus mainly on manufacturing, mining, and producing semiconductors and other finished goods while slowly shifting to providing services. The income classification of countries is based on gross national income per capita (in US dollars [USD]) using the World Bank Atlas method; the results show that Southeast Asia is the poorest area in the Asia Pacific region, with most countries having a GDP per capita less than USD $10 000 (Table 1; World Bank 2017a).

Because the Asia Pacific region is home to more than half of the world's population, water security, clean water availability, and water sanitation are consistently great challenges (Asian Development Bank 2016). The situation is especially alarming in the poorest area of the region, Southeast Asia, where agriculture and manufacturing activities are the main industries that have a high demand for water resources and at the same time generate pollution. Although water security problems have improved over the past decades, there remains much room for improvement in household water supply and sanitation. For example, there are approximately 1.7 billion people living in areas without proper water and wastewater treatment (Eco‐business 2011; Asian Development Bank 2016). Most countries are only equipped with septic tanks or pit latrines for collecting wastewater, and discharge to nearby water bodies occurs without any treatment, especially in rural areas (Table 1). Biological wastewater‐treatment facilities are mainly installed in major cities. Installation of advanced facilities is rare in Oceania, where countries usually directly discharge used water to the marine environment because of the low population density. However, the mixed usage of drainage systems and lack of water infrastructure may lead to the outbreak of water‐related health diseases such as cholera (World Health Organization 2020). Therefore, the development of advanced water infrastructure, improvement of wastewater facilities, and management of environmental and human health hazards associated with poor water quality are urgently needed in this region. In 2013, only 32% of sewage in Asia received treatment (Sato et al. 2013), though the technologies employed and the associated effectiveness vary among countries, which further highlights the water‐ and waste‐management challenges in this part of the world.

Besides water insecurity associated with poor quality, biodiversity conservation is another great challenge in Asia. This region is rich in biodiversity, with the most diverse coral reefs in Southeast Asia and numerous unique species found on various isolated islands, especially in Oceania (United Nations Environment Programme 2016; Hughes 2017; International Maritime Organization 2017). Approximately 70% of the world's species are found in 12 countries: Australia, Brazil, China, Colombia, Costa Rica, the Democratic Republic of Congo, Ecuador, India, Indonesia, Madagascar, Mexico, and Peru, 4 of which are located within the Asia Pacific region (Hood 2010). However, many species in this region are under threat that is closely related to anthropogenic factors such as increasing population, its associated increase in pollution of water bodies, urbanization, deforestation, illegal harvesting, illegal trading, as well as climate change. Of 36 recognized biodiversity hotspots where >70% of the original habitat has been lost, 14 are found in the Asia Pacific region (Hood 2010; Critical Ecosystem Partnership Fund 2016).

Given the high population density, rapid urbanization, and industrialization in the region, especially in Southeast Asia, there are inevitably increasing chemical contaminants in water, air, and soil/sediment. In addition, global chemical production is increasingly centered in Asia (United Nations Environment Programme 2019); for example, China, Japan, and Korea are 3 of the top 5 countries for global chemical sales (European Chemical Industry Council 2020). Pollution problems are further intensified by a lack of environmental infrastructure to control the release of pollutants at the source and cleanup in many developing nations in this region. Critical global hotspots of biodiversity in tropical Asia are facing multiple threats including chemical pollution. Researchers and regulators in environmental quality management play essential roles in revealing the environmental fate of these chemicals, evaluating their environmental risks, and providing science‐based solutions to mitigate and minimize their negative impacts to the environment and human health. However, identifying which specific priority research needs should be addressed to advance more sustainable environmental quality in Asia has remained elusive.

The Global Horizon Scanning Project (GHSP) was launched to identify important environmental quality research needs around the world, by transparently engaging diverse disciplines working in the government, academia, and business sectors (Brooks et al. 2013). With the support of the Society of Environmental Toxicology and Chemistry (SETAC), the GHSP has been successfully accomplished in the Australasian region of Oceania, Europe, North America, and Latin America; and its priority research questions have been published (e.g., Furley et al. 2018; Van den Brink et al. 2018; Fairbrother et al. 2019; Gaw et al. 2019). As part of the GHSP, we invited members of the SETAC Asia Pacific Geographic Unit to recommend priority research questions for Asia in relation to chemical risk management with consideration of the aforementioned demographic characteristics, economic development, and geographic features of the Asia Pacific region. Because a similar HSP exercise was recently conducted for Oceania (Gaw et al. 2019), we specifically focused on Asia. This Asian HSP exercise aimed to identify the top priority research questions in the region through bottom‐up stakeholder engagement. Outcomes of this HSP were thus intended to beneficially inform the researchers, funding agencies, and regulatory authorities regarding key research priorities and knowledge gaps in the region.

## METHODS

Here, we employed a transparent and inclusive approach and followed a well‐established social science process in selection of the research questions (Sutherland et al. 2011). In 2016, members of the SETAC Asia Pacific Geographic Unit (~550 members from academia, government, and business) were invited via email to submit priority research questions to advance toward more sustainable environmental quality using an online platform. Like other regional HSP exercises, members were informed about how to develop an ideal priority question (Sutherland et al. 2011), which should address important knowledge gaps, be answerable through a realistic experimental design, be answerable within a 5‐yr period if supported with sufficient research funding (e.g., €5 million), cover a spatiotemporal scale that could realistically be addressed by a research team, not be answerable by “it all depends” or “yes” or “no,” and contain a subject, an intervention, and a measurable outcome. In addition, the questions were solicited to be research‐oriented, rather than policy‐based, while the spatial scale had an Asian focus, within a global context. Thus, this workshop format was more inclusive than traditional formats.

After gathering the submitted questions, the project team reviewed them and removed any duplicated questions and responses outside the scope of the exercise. Then, we put forward a list of 113 questions for further deliberation at the Asia Horizon‐Scanning Workshop, which was organized in conjunction with the 2016 SETAC Asia Pacific meeting in Singapore. The workshop participants were living or working in the Asia Pacific region. They included tripartite (74% academia, 17% business, 9% government), geographic (from 14 different countries), disciplinary and gender (60% males, 40% females) diversity. Such diversities of the members who originally submitted questions and workshop participants were thus consistent with previous horizon scanning efforts in other geographic units. During this 1‐d Asia Horizon‐Scanning Workshop, there were 2 breakout sessions, each session having 3 concurrent theme groups. Hence, 6 themes were initially arranged for the group discussion purposes, including 1) tools for improving risk assessment; 2) multiple stressors and mixtures; 3) contaminants of emerging concern; 4) risk assessment, regulation, and guidelines; 5) environmental chemistry and engineering; and 6) spotlight on Asia. The submitted questions were assigned to the 6 themes according to relevance and then deliberated by participants with multidisciplinary expertise from the government, academia, and industry sectors in Asia. To generate the priority research questions, the workshop participants further identified 2 to 5 priority research questions in each theme, and then the combined list of the research questions was further discussed, prioritized, and endorsed at a final plenary session, consistent with methods of other GHSP workshops (Furley et al. 2018; Van den Brink et al. 2018; Fairbrother et al. 2019; Gaw et al. 2019). Finally, the selected priority questions were grouped into 4 overarching themes based on their contexts and relatedness.

## RESULTS AND DISCUSSION

From the 113 questions, 23 priority research questions for Asia were identified during the Singapore Workshop (Table 2). These questions were broadly identified within 4 overarching themes: 1) environmental fate and risks of chemical contaminants; 2) advanced technologies for understanding and predicting toxicities and environmental risks of chemical contaminants; 3) issues of multiple stressors; and 4) sustainability, food safety, and green chemistry.

**Table 2:** The 23 priority questions identified by the Asian Horizon Scanning Project (HSP) among 4 themes^a^

No.		Europe	Latin America	North America	Oceania	Total
Theme 1: Environmental fate and risks of chemical contaminants						
1	How do we develop broad screen analytical methods integrating nontarget directed analysis for identifying key chemical stressors responsible for observed toxicity?	✓		✓	✓	3
2	How do we develop methods to identify and quantify nano‐ and microplastics in different environmental compartments (water, sediment, soil, biota) associated with potential toxicity or interactions with other contaminants?		✓	✓		2
3	What are the terrestrial and aquatic risks of atmospheric contaminants in Asia?					0
4	How can we improve methods to classify, identify, and separate nanomaterial contaminants from their bulk counterparts and differentiate effects caused by nanomaterials in the environment?		✓			1
Theme 2: Advanced technologies for understanding and predicting toxicities and environmental risks of chemical contaminants						
5	How can we better use field data and incorporate new big data (e.g., ecological genome) approaches for improving ecological risk assessments and decision‐making?	✓		✓	✓	3
6	How can we develop and advance laboratory (e.g., in vitro, in vivo, analytical) and theoretical (toxicokinetic, toxicodynamic) approaches to understand (prospective, retrospective) adverse outcomes of complex chemical mixtures (e.g., pesticides, surfactants, medicines, metals)?	✓	✓		✓	3
7	How we can improve the current approaches to assess and manage risks of micropollutants and emerging contaminants?	✓		✓	✓	3
8	How can we integrate high‐throughput screening with next‐generation computational toxicology tools to support hazard and risk assessment of individual chemicals and complex mixtures?	✓		✓	✓	3
9	How can we develop advanced biological tools to better understand and predict toxic mechanisms and interactions across species in multiple highly biodiverse compartments for risk assessment and management of chemical contaminants in Asia?		✓	✓	✓	3
10	How can we analyze big data and develop effective risk‐communication approaches (e.g., report card system, real‐time reporting) for environmental status (e.g., ecosystem functions and services)?	✓		✓		2
11	How can we use new developments in nanoscience and nanotechnology to advance ecotoxicological research?					0
Theme 3: Issues of multiple stressors						
12	How can we strengthen the environmental quality criteria system (e.g., water, sediment, soil, air) to adequately protect ecosystems that are experiencing multiple stressors and changing climate?		✓		✓	2
13	What are the influences of changing landscapes and climate change on the resilience of terrestrial and aquatic ecosystems, and how do we measure the ecological endpoints with reference to chemical pollution?	✓	✓	✓	✓	4
14	How can we develop an integrative and effective framework (e.g., environmental policy, green technologies) to manage nutrient loading and associated hypoxia in Asia?			✓		1
15	How will changes to physicochemical characteristics (e.g., salinization/ion imbalance, pH, temperature, hypoxia attributable to enrichment) alter the bioavailability and effects of chemical stressors in the environment?			✓	✓	2
16	How can we prioritize and apportion chemical stressors in complex scenarios to guide restoration efforts?	✓		✓	✓	3
17	How can we identify adverse impacts of multiple stressors in the field to biodiversity (including multigenerational, evolutionary, and developmental), ecosystem services, and human health?	✓	✓	✓		3
18	To what extent is seawater pH in Southeast Asia impacted by terrestrial inputs (e.g., organic carbon, nutrients, other anthropogenic sources such as mining), how are these inputs changing as a result of human activities (including CO_2_), and how does this affect vulnerable coastal ecosystems such as coral reefs?					0
Theme 4: Sustainability, food safety, and green chemistry						
19	How can we develop new technology and promote green chemistry for enhancing reuse of waste and preventing environmental impacts?	✓	✓	✓	✓	4
20	Given increasing population growth and per capita demand for seafood in Asia, how can we develop sustainable aquaculture practices while protecting environmental quality, particularly in coastal waters?	✓				1
21	How can we develop innovative solid waste‐management programs to protect environmental quality, particularly in rural areas of less developed regions in Asia?				✓	1
22	What is the extent of antibiotic pollution in the environment and associated risks of antibiotic resistance in rural and urban regions of Asia?		✓	✓	✓	3
23	How can we develop sustainable development frameworks (e.g., green chemistry) to address, balance, and manage the production (e.g., food production, forestry) and protection of ecosystem services?	✓	✓	✓	✓	4
Total		12	10	15	14	

^a^ If a similar question was reported by the HSP in another region (i.e., Europe, Latin America, North America, and Oceania), it is indicated with a tick.

### Environmental fate and risks of chemical contaminants

Two types of information are prerequisites for environmental risk assessment of chemical contaminants: 1) measured or predicted environmental concentrations (PECs) of the chemical of concern and 2) its predicted‐no‐effect concentration (PNEC), which is derived from available toxicity data (Lin et al. 2005; Lin and Meng 2009; Leung et al. 2014, 2018; Zhou et al. 2019). Four priority research questions related to the detection and quantification of various emerging chemical contaminants in different environmental compartments (i.e., PECs) and how to relate observed toxicity to PECs of individual chemicals in a mixture.

### How do we develop broad screen analytical methods integrating nontarget directed analysis for identifying key chemical stressors responsible for observed toxicity? (Q1)

Asia is one of the fastest‐growing areas in the world. In addition to intensive agriculture practices to feed the high population in this region, rapid urbanization and industrialization pose a high stress to the environment. As a result, the ecosystems, including humans, in this region have been, and still are, continuously exposed to a complex mixture of legacy and emerging contaminants which arise from a variety of sources, such as domestic sewage, industrial wastes, agriculture runoff, and aquaculture practices. The occurrence of contaminant “cocktails” in the environment poses potential ecological risks, yet these risks are inadequately understood, especially in developing nations in Asia. While evaluating the ecological risk caused by individual contaminants in aquatic ecosystems has been relatively well developed, especially for common chemical contaminants (e.g., the AIST‐Multi‐purpose Ecological Risk Assessment and Management Tool; https://en.aist-riss.jp/softwares/5511/), tackling multiple stressors in a complex system at the same time is an essential step forward (Escher et al. 2020). In this context, it is imperative that more holistic methods to assess the risk in ecosystems are developed, and both the key stressors and the causal relationships between the stressors and ecological responses are identified. Recent studies suggest that the observed toxicity is driven mainly by a few chemicals, although multiple contaminants are present in the environment (e.g., Stehle and Schulz 2015). Before effective measures to mitigate the risk in ecosystems can be implemented, it is important to identify the driving agents for the observed toxicity within the systems. Traditional ecological risk evaluations have typically compared chemical concentrations in environmental media to available thresholds (i.e., PNECs) to define risk (Maruya et al. 2016), and the target chemical analysis–based methods are in many circumstances unable to prioritize key toxicants in complex mixtures. Fortunately, there is growing awareness of the importance of the challenges, expressed in a growing body of research on nontarget chemical analysis (Leung 2018; Moschet et al. 2018). Although the development and harmonization of sample databases for authentication are urgent needs for the application of nontarget analysis approaches, the development of sophisticated instruments in recent years, particularly a variety of high‐resolution mass spectrometers, along with the integration of computational chemistry and data science, brings hope that we will see substantial progress in the field of nontarget analysis. With such analytical advancements, a combination of toxicity identification evaluation and effect‐directed analysis can provide a more holistic understanding of adverse effects from both target and nontarget contaminants (Li et al. 2018b).

### How do we develop methods to identify and quantify nano‐ and microplastics in different environmental compartments (water, sediment, soil, biota) associated with potential toxicity or interactions with other contaminants? (Q2)

Asia is one of the leading regions for plastics production, accounting for nearly half of the world's production (Wu et al. 2017). Accordingly, the input of plastic wastes from East Asia into the adjacent ocean was estimated as the highest in the world (Jambeck et al. 2015). In the field, plastic wastes are gradually broken into small pieces and finally microplastics (<5 mm) and even nanoplastics (in nanometers). It is important to note that the sizes of nano‐ and microplastics overlap with naturally occurring particles such as sediment clay (<2 μm), silt (2–50 μm), and sand (0.05–2 mm; Connors et al. 2017). Therefore, it is necessary to develop and standardize effective methods to differentiate nano‐ and microplastics from naturally occurring particles and quantify their occurrence in different environmental compartments, including water, sediment, soil, and biota. Moreover, there may be a cocktail of organic chemical contaminants, which are absorbed into and/or adsorbed onto the micro‐ and nanoplastics, could pose harmful effects to living organisms after ingestion of these contaminated particles (Ziccardi et al. 2016). Understanding the interactions among microplastics and associated chemicals is essential to evaluate the potential risk related to plastic debris.

### What are the terrestrial and aquatic risks of atmospheric contaminants in Asia? (Q3)

As discussed in the *Introduction*, a great proportion of the human population resides in Asia (Table 1). Extensive human activities associated with the high population density in this region have resulted in a growing demand for various resources as well as a constant release of pollutants into the environment. As such, a variety of contaminants have been detected in different environmental compartments, such as air, soil, water, and sediment. Most studies on the occurrence and risk of contaminants in the environment are restricted to a single compartment, but the environment is not an assembly of isolated compartments. Therefore, it is important to understand the movement of contaminants across different environmental compartments, and a variety of multimedia fate models have been developed and increasingly used for predicting the fate and transport of legacy and emerging contaminants (Cowan et al. 1995). Air pollution is a severe problem in Asia, especially in India and China. Although extensive studies have been conducted on ambient air pollution and its associated health issues in this region as well as the transport of airborne contaminants across country boundaries (Zhong et al. 2012), little is known about how these atmospheric contaminants may affect the terrestrial and aquatic ecosystems via precipitation processes. Soil and sediment are important sinks for a suite of contaminants, and as a result, contaminants in air may eventually end up in soil and sediment, potentially causing adverse effects to organisms dwelling in these compartments. Given that air pollution is a major concern in Asia, a high priority should be given to research studies that incorporate risk assessment and multimedia fate modeling to reveal the composition, fate, and transport of air pollutants (Li et al. 2014; Lu et al. 2018).

### How can we improve methods to classify, identify, and separate nanomaterial contaminants from their bulk counterparts and differentiate effects caused by nanomaterials in the environment? (Q4)

A great number of laboratory experiments have been conducted for assessing adverse outcomes of nanomaterials (Lai et al. 2018). Unfortunately, the key drivers of toxicity in these assessments are not clear in some cases, and the observed effects may be caused by their bulk counterparts and released ions or degradants instead of the nanomaterials themselves. Therefore, the selection of appropriate methods to identify nanomaterials and differentiate their toxicity from bulk counterparts is imperative (Geitner et al. 2020). Such a finding is partially attributable to the fact that there has been increasing evidence to show that many nanomaterials are not consistently more toxic to living organisms when compared with their bulk counterparts (Wiesner 2019). Hence, current regulations on bulk chemicals may already offer adequate environmental protection from nanomaterials (Lai et al. 2018). Nonetheless, the movement of nanoparticles including nanoplastics within organisms including humans, their long‐term health implications, and their interaction with other coexisting chemicals are still largely unknown (see Question 2). To better understand the environmental fate and potential environmental risk of nanomaterials, we will need to tackle the major challenges in the field of nanotoxicology including how we can isolate target nanoparticles from environmental samples, accurately monitor and quantify them, characterize their physicochemical properties (e.g., aggregation size, zeta potential, ion dissolution), and link these results to their observed toxicity in living organisms (Lai et al. 2018).

With rapid advancements in analytical instrumentation and DNA sequencing, we have been entering a new era of high‐throughput omics in recent years. Such technologies enable us to obtain a large amount of data on biological responses to chemicals in terms of gene expression (transcriptomics), protein expression (proteomics), and metabolite profiles (metabolomics; Leung 2018). We can now use these omics' approaches to uncover the toxic mechanisms of chemicals and their mixtures at the molecular and ecosystem levels. However, technological breakthroughs will be dependent on further development of complementary mega‐database and better bioinformatics for handling such large data sets. Under this central theme, there are 7 priority questions.

### How can we better use field data and incorporate new big data (e.g., ecological genome) approaches for improving ecological risk assessments and decision‐making? (Q5)

Because the current paradigm of ecological risk assessments of chemical contaminants is heavily dependent on laboratory‐driven toxicity data for individual chemicals and individual culturable species, there is a high uncertainty associated with use of such laboratory data to derive and extrapolate PNECs for the protection of actual ecosystems and their resident biota, hence resulting in high safety margins (Lin et al. 2005; Lin and Meng 2009; Leung et al. 2014, 2018; Merrington et al. 2014). Field data can be used to cross‐check the validity of the laboratory‐based PNECs (Leung et al. 2005) and provide direct evidence on the ecological process and status of an ecosystem (e.g., soils, rivers, lakes, estuaries) that are potentially impacted by toxic substances and, under some circumstances, constitute a line of evidence in guideline determination itself (Cormier and Suter 2013; Australian and New Zealand Governments 2018). Such applications have been historically restricted by the limitation of field survey technologies (Yang et al. 2018), but that situation is rapidly changing (Australian and New Zealand Governments 2018).

The advancement of ecogenomics approaches provides an open system to evaluate the ecological structure and function of an ecosystem in a high‐throughput fashion (Chariton et al. 2016; Leung 2018; Zhang et al. 2018). For example, meta‐barcoding of environmental DNA (eDNA), as a new biological survey method (Deiner et al. 2017), can be used to monitor the phylogenetic diversity of organisms by detecting species‐specific target DNA from water (Yang et al. 2017; Li et al. 2018a) and sediment samples (Xie et al. 2018a, 2018b). In recent years, eDNA technologies have been rapidly developed, primarily focusing on method development and application in the field. To fully apply their potential in environmental impact assessment, some uncertainties must be addressed including the lack of indication of abundance of each specie and spatial uncertainties due to eDNA movement (e.g., flow dynamics in rivers). Fortunately, there are ongoing research efforts to solve these issues (e.g., Altermatt et al. 2020).

Meta‐transcriptomics and meta‐metabolomics can analyze the functional and metabolic diversity of species within naturally occurring assemblages of organisms (e.g., communities; Grossmann et al. 2016). Integration of field‐based mesocosms and ecogenomics can provide an excellent platform to assess the chemical‐induced alteration on biodiversity, community composition, and ecosystem function using a tree‐of‐life approach (Li et al. 2018a; Yang et al. 2018). For example, eDNA meta‐barcoding technologies can be applied in assessing community‐level effects triggered by toxic stressors. Firstly, samples can be collected from different sites along a stress gradient and the species composition obtained by meta‐barcoding. Secondly, response patterns of different taxa can be classified to different groups, for example, sensitive, tolerant, and nonaffected groups. Field‐based species sensitivity distributions (f‐SSDs) are developed by the 50% abundance concentration of the sensitive taxa (Leung et al. 2005). Finally, an ecological source‐to‐outcome pathway can be built by a linear connection among aggregate exposure pathways (Teeguarden et al. 2016), adverse outcome pathways, f‐SSDs, altered community structures, and altered ecosystem services. Such a novel field‐based approach will require further validation through field studies in different habitats in Asia. Alternatively, a multivariate approach comparing reference to successive exposure gradient classes may also derive a dose–response relationship (Chariton et al. 2016; Australian and New Zealand Governments 2018). Nonetheless, it is important to note that most of the published and current relevant field‐based studies are conducted as small‐scale case studies and for academic research purposes and have yet to be commonly implemented for large‐scale studies and widely adopted by environmental authorities. Thus, it remains a scaling challenge for developing an easy‐to‐use and reliable field‐based approach to be adopted by environmental authorities in routine environmental risk assessment.

### How can we develop and advance laboratory (e.g., in vitro, in vivo, analytical) and theoretical (toxicokinetic, toxicodynamic) approaches to understand (prospective, retrospective) adverse outcomes of complex chemical mixtures (e.g., pesticides, surfactants, medicines, metals)? (Q6)

National‐scale biomonitoring programs such as the National Health and Nutrition Examination Survey of the United States and the Korean National Environmental Health Survey show that humans are exposed to a myriad of chemicals at the same time and suggest that other living organisms in ecosystems may not be an exception. Therefore, understanding and managing adverse outcomes of complex mixtures is one of the grand environmental health challenges of modern societies including those in Asia (Carusi et al. 2018). Mixtures can be as simple as a binary mixture but also can include a huge number of components. Diesel engine exhaust and oil spills are examples of extremely complex mixtures. Traditionally, mixture interaction was assumed to be represented in an “additive” way, that is, dose or response addition, if the components in the mixture have the same or similar modes of action or responses (Kar and Leszczynski 2019). The National Research Council of the United States recommended that cumulative risk assessment for mixtures be conducted for chemicals not only with the same modes of action but also with the same type of health outcomes (National Research Council 2008).

For mixtures for which modes of action or adverse outcomes of the components are well characterized, dose or response addition can be an option to estimate the toxicity. However, often, the complexity of the mixture composition and a lack of toxicological information can be obstacles. For example, environmental health issues of per‐ and polyfluoroalkyl substances (PFAS) have become of growing interest, but characterizing their toxicological properties in a timely and cost‐effective way is very challenging because there are more than 4000 PFAS of possible concern (Lim 2019).

Recently, Zhang et al. (2018) proposed a high‐throughput functional genomic screening measure in combination with a cell‐ and a fish embryo–based reduced transcriptomics for mechanistic research on toxicological effects of chemicals. With the reduced gene set for high‐throughput transcriptomics, this conceptual framework has promise in addressing mixtures of multiple chemicals, even including hydrophobic chemicals (Vergauwen et al. 2015; Balik‐Meisner et al. 2019). This approach can identify toxicological modes of action of chemicals employing multiple biological organizational levels ranging from the molecular to the cellular and individual (or even community) levels, in a high‐throughput manner, which enables grouping of multiple chemicals based on the toxicity pathway or the toxicological modes of action or possible toxicological responses (Xia et al. 2020). Once chemicals are grouped, validation can be performed using in vivo high‐throughput platforms (e.g., Kithcart and MacRae 2017; Meng et al. 2020). As a high‐throughput in vivo screening tool, fish embryos are a promising model because of their utility in both ecotoxicological and environmental health studies (Bambino and Chu 2017) and their advantage of less restrictive guidelines in terms of experimental use (European Parliament 2012). Depending on the similarity of the mode of toxic action, additive joint toxic action can be applied to estimate the mixture toxicity (US Environmental Protection Agency 2000b).

This approach can also be applied to the mixture as it is, especially when the mixtures of concern have a complex composition. Because whole effluents are considered to be “stressors” and measured for ecotoxicity for discharge permit (see the whole‐effluent toxicity test of the United States [US Environmental Protection Agency 2000a] and Japan [Ministry of Environment of Japan 2019] and the ecotoxicity management policy of Korea [Ministry of Environment of Korea 2015]), one can consider specific complex mixtures as individual chemicals and estimate their adverse outcomes. Such mixtures may include commercial products with diverse chemical composition (e.g., surfactants or pesticides) or environmental samples of complex mixture (e.g., fine particulates in air, dissolved organic fractions of water).

### How we can improve the current approaches to assess and manage risks of micropollutants and emerging contaminants? (Q7)

Micropollutants are synthetic substances that appear in concentrations usually <1 μg/L in natural waters (Fuhrmann 2012; Stamm et al. 2016). Emerging contaminants are both human‐made and naturally occurring chemicals that have not commonly been monitored in the environment. In some cases, some of these chemicals are or have been monitored, but we do not know what they might do to the environment (i.e., lack of effects data). Because the number of chemicals used in modern society has been increasing and many of these emerging chemicals may cause serious ecological and health risks, improving approaches to assess and manage the risks of micropollutants and emerging contaminants is warranted.

Because it is not feasible to assess and manage all micropollutants and emerging contaminants, identification and prioritization of chemicals that have higher risks and require proper management are crucial first steps. Although a systematic approach should be established to identify priority chemicals and subsequently develop appropriate management plans, most Asian countries do not have such a system. Taking Korea as an example, when a new micropollutant is detected in the water at a level that may cause health risk, it is required by regulation to identify the sources of the release and to develop and implement management systems for the pollutant, including mitigation measures (Ministry of Environment of Korea 2009). However, a systematic procedure that can be applied to identify new micropollutants is not available in Korea. Identification of new chemicals of concern generally relies on monitoring programs designed for specific purposes not necessarily related to prioritization. For example, the Ministry of Environment of Korea designated 3 PFAS compounds, i.e., perfluorooctane sulfonate, perfluorooctanoic acid, perfluorohexane sulfonate, as watch‐list chemicals for drinking water in 2018, after detecting these chemicals in greater frequency in drinking water treatment plants in a government‐funded study (Ministry of Environment of Korea 2017). The Korea example exemplifies the urgent need for a more systematic approach to identify candidate chemicals in Asia.

Because micro‐ and emerging contaminants often have not been frequently measured and/or are present at low levels, advances in chemical analytical methods are necessary. Recent advances in analytical chemistry which include techniques related to nontarget analysis will be useful eventually for this effort (Schymanski et al. 2015; see Question 1). Another challenge lies in the lack of toxicological information for these micro‐ and emerging contaminants. Most of the available toxicological information is limited to traditional toxicological endpoints outlined by regulatory or international entities, for example, Organisation for Economic Co‐operation and Development (OECD) test guidelines. In some cases, such traditional toxicological information may not capture important modes of toxicological action of these chemicals. Therefore, developing high‐throughput screening measures in combination with advanced bioanalytical models is warranted. For example, the toxicological priority index, which supports a rational prioritization of chemicals using ToxCast data, may be considered one possible method for this purpose (Reif et al. 2010).

In contrast to the top‐down approach, a bottom‐up approach that starts from toxicological responses can be another option. This bottom‐up approach can be described as an ecological version of epidemiological investigation (“eco‐epidemiology”) that helps identify a fraction (or group) of chemicals that are responsible for a given ecological adverse outcome. This eco‐epidemiological approach can be coupled with toxicological approaches such as effect‐driven analysis and can be used to identify micro‐ and emerging contaminants that warrant follow‐up assessment and management (Bornstein et al. 2014). Hence, this approach may supplement the top‐down approach, which can miss an important chemical(s) that may cause adverse outcomes.

### How can we integrate high‐throughput screening with next‐generation computational toxicology tools to support hazard and risk assessment of individual chemicals and complex mixtures? (Q8)

As discussed, Asia has experienced unprecedented increases in population and economic growth during the past several decades, resulting in the release of increasingly diverse chemicals into the market and the environment. To screen and assess the hazard (and risk) of these chemicals, traditional approaches need to be significantly improved. Computational toxicology tools have been considered as one of the promising alternative approaches for hazard and risk assessment of chemicals (Mangiatordi et al. 2016). One good example is the OECD Quantitative Structure–Activity Relationship (QSAR) Toolbox, which is one of most widely used computational tools for prediction of chemical toxicity including read‐across (Schultz et al. 2018). Because of the complexity of toxicological mechanisms and biological responses, a more integrated approach employing advanced data processing (Meng and Lin 2007), machine learning techniques (Takada et al. 2019), and omics data becomes necessary (Kiani et al. 2018). Otherwise, such computational models could be misused and often lead end users to elusive or underprotective results (Mangiatordi et al. 2016).

Therefore, integrating next‐generation computational toxicology tools with high‐throughput screening measures is essential. As a result of significant advances in omics technologies, image techniques, and automated robotic platform techniques in recent decades, testing a growing number of chemicals for toxicity in a high‐throughput manner becomes more feasible (Kiani et al. 2018). The Toxicology in the 21st Century program is a good example of a collaborative approach among several agencies of the United States to better predict toxic chemicals using robotics in high‐throughput screening (Dox et al. 2006).

Direct application of the knowledge obtained from computational and high‐throughput screening measures to hazard or risk assessment of chemicals will eventually uncover significant uncertainties, including taxa applicability and mechanistic probability, to name a few. Efforts to address such uncertainties through scientific and methodological advances are essential, even though it may not be possible to completely remove the uncertainties. In addition, building consensus among regulatory, industrial, and societal stakeholders on the values and limitations of these computational and high‐throughput screening measures is very important.

### How can we develop advanced biological tools to better understand and predict toxic mechanisms and interactions across species in multiple highly biodiverse compartments for risk assessment and management of chemical contaminants in Asia? (Q9)

Chemical contaminants in the environment can generate adverse effects at all levels of biological organization from the molecular to the population, community, and ecosystem levels (Zhang et al. 2018). To improve the understanding and prediction of the mechanisms of adverse outcomes in a multiple, highly biodiverse ecosystem, advanced biological tools should be developed to capture the changes across the multiple levels of biological organization, with a particular focus on indigenous species and communities in the local environment of concern (i.e., site‐specific).

Effect‐based methods, compiling a battery of in vitro assays and apical bioassays (e.g., with fish embryos, daphnia, and algae) and covering a range of well‐described modes of action and diverse taxa (Escher et al. 2014; Altenburger et al. 2015), can provide both diagnostic and monitoring information to establish the likelihood of impacts of chemical contamination (Brack et al. 2015). Omics technologies, if well applied, have the potential to enable qualitative and quantitative measurement of changes in different biological organization scales through molecular, cellular, tissue, individual, population, and community levels and thus provide a historic opportunity to transform our knowledge of the consequences of the exposure of toxic substances in the environment (Leung 2018; Zhang et al. 2018). Recent omics advances, such as reduced transcriptomics (Xia et al. 2017; Wang et al. 2018) and eDNA meta‐barcoding (Deiner et al. 2017; Yang et al. 2017), not only generate new knowledge regarding mechanisms of chemical toxicity and their environmental effects and improve the relevance and immediacy of laboratory toxicological assessment but also can provide a wholly new paradigm for ecotoxicology by linking ecological models to mechanism‐based, systems biology approaches. Future chemical risk assessment and management in Asia can benefit from focusing on the site‐specific protection goals, establishing an integrative risk assessment framework, and efficiently utilizing the new approaches as described herein.

### How can we analyze big data and develop effective risk‐communication approaches (e.g., report card system, real‐time reporting) for environmental status (e.g., ecosystem functions and services)? (Q10)

Rapid and reliable ecological monitoring and prediction of the trend of ecological changes are of great value to the protection of ecosystems. Recent advances in environmental omics (e.g., meta‐barcoding), remote sensing, and other interdisciplinary technologies not only allow sensitive automated biomonitoring of terrestrial and aquatic ecosystems in high spatial and temporal resolution at larger scales but also enable more accurate and cost‐effective detection of ecological changes (Gibson et al. 2015; Dafforn et al. 2016; Bohan et al. 2017).

Effective utilization of big data generated by these novel approaches relies on efficiently transforming and extracting the core information and key messages into risk communication on the environmental status (Gibson et al. 2015). In future, development of simple, convenient, and standardized operations will be important to make these technologies more accessible and facilitate effective risk communication. Also, a combination of multiple approaches, such as integrating eDNA meta‐barcoding and remote sensing with machine learning, will revolutionize our understanding of ecosystem changes. Finally, in addition to the “report card” system, the citizen science risk community tools (e.g., public participatory geographical information systems) would be another effective approach to engage the general public to address environmental concerns (Jiao et al. 2016; Deiner et al. 2017).

### How can we use new developments in nanoscience and nanotechnology to advance ecotoxicological research? (Q11)

In the past 2 decades, advancements of nanoscience and nanotechnology have led to numerous innovations and establishment of novel technologies for various industries including the environmental sector. For example, specially synthesized magnetic nanoparticles can be used to break down and remove micropollutants from sewage effluents (Hu et al. 2005; Tang and Lo 2013). Because of advances in nanoscaled functional materials, many sensors for environmental monitoring can be more compacted and reduced in size with higher specificity and sensitivity (Su et al. 2012). Therefore, participants of this Asia HSP noted that there is great potential for developing and applying various nanotechnologies to advance ecotoxicological research, and this area remains relatively underexplored. Other possibilities may include 1) the development and use of fluorescent nanoparticles as bioimaging probes (i.e., labels) for studying the fate and toxicity mechanisms of chemical substances within cells and organisms (e.g., Bhunia et al. 2013), and 2) the innovation and integration of lab‐on‐chips and biochemical sensors to study the toxicity of single chemicals and chemical mixtures with cells or small aquatic organisms (i.e., downscaled experimental setups; Figeys and Pinto 2000). The latter would enable high‐throughput and rapid analysis and promote sustainability by substantially reducing the amount of chemicals being used in toxicity tests.

In the natural environment, chemical contaminants often exist in mixtures while their physicochemical properties and toxicities are governed by environmental conditions (e.g., temperature, solar radiation, and moisture in soils; water temperature, conductivity, and pH in aquatic environments; Wang et al. 2016, 2019b; Leung et al. 2018). We agree that it is very important to study the impact of chemical contamination while considering the coexistence and combined effects of multiple stressors. Multiple stressor issues become more apparent and pressing under anthropogenically driven climate changes, including warming and heatwaves, alteration in the patterns of rainfall and typhoons, and acidification of water bodies. There are 6 questions relevant to the theme of multiple stressors.

### How can we strengthen the environmental quality criteria system (e.g., water, sediment, soil, air) to adequately protect ecosystems that are experiencing multiple stressors and changing climate? (Q12)

Setting protective PNECs or environmental quality benchmarks (EQBs) of chemical contaminants is a prerequisite step in environmental risk assessment and management of environmental quality (Leung et al. 2014, 2018; Merrington et al. 2014; Zhou et al. 2019). There are some obvious shortcomings in the current system for setting EQBs in Asia. First, much of the available toxicity data were generated using temperate or cold‐water species, but most Asian countries are situated in the tropics, where toxicity data are lacking (Kwok et al. 2007; Wang et al. 2014, 2019a; Mooney et al. 2019). Because of the insufficiency of tropical toxicity data, Asian countries in the tropics often made use of temperate data to derive their EQBs or directly adopted the EQBs from North America or Europe for managing their environmental quality (Kwok et al. 2007), while unknown margins of protection are often addressed by application of safety factors. This results in an unknown margin of protection for tropical ecosystems in Asia. Second, the current EQB derivation system mainly deals with chemical toxicity in standard representative environmental conditions (e.g., a fixed average temperature) without consideration of the influence of seasonal and annual changes of environmental conditions (Zhou et al. 2014; Leung et al. 2018). As such, the derived EQBs may not be protective under extreme climate conditions which are influenced by global climate change. For instance, Wang et al. (2019b) discovered that extremely high or low temperatures can exacerbate chemical toxicity to freshwater organisms. Therefore, there is an urgent need to develop a region‐specific EQB derivation system, including identification of indicator species for testing and risk assessment, with a view to generating more accurate PNECs for protecting the ecosystems in Asia.

Furthermore, current EQB derivation systems around the world mainly deal with the derivation of PNECs for individual chemicals without consideration of chemical mixtures nor the co‐occurrence of other multiple stressors (e.g., temperature), an issue not restricted to Asia. Recently, Mu et al. (2018) made an attempt to predict the toxicities and PNECs of various metals in different marine environments around the globe by integrating QSAR models with temperature‐ and salinity‐based SSD approaches. This innovative study, as an example, paves the way for developing region‐specific toxicity prediction models and hence strengthening the EQB derivation system to enable the derivation of region‐ and site‐specific PNECs for a wider range of chemical contaminants.

### What are the influences of changing landscapes and climate change on the resilience of terrestrial and aquatic ecosystems, and how do we measure the ecological endpoints with reference to chemical pollution? (Q13)

This is an overarching question regarding the interplay among 3 different stressors—land use, climate change, and chemical pollution—which can be applicable not only to Asia but also around the globe. The biological communities and ecosystem functions of an aquatic ecosystem can be altered by a combination of these 3 interrelated factors, while at the same time, the effects threshold of a chemical contaminant to the aquatic ecosystem might be modified by the changing environmental condition. For example, conversion of a natural wetland into a city with impervious (concrete) surfaces would increase the discharge of pollutants via surface water runoff (He et al. 2020), while under global warming the bioavailability and toxicity of chemicals could also be increased (Zhou et al. 2014; Wang et al. 2019b). Both of these changes might eventually pose a higher risk to aquatic organisms. Such a complex question requires a sophisticated method to disentangle the impacts of multiple stressors and the ecosystem response. Chariton et al. (2016) recommend a conceptual model to fulfill this purpose, encompassing the collection and analysis of an integrated data set on biological composition and function, biotic and abiotic stressors, and other information available in a geographical information system. Through advanced statistical analyses, it is possible to identify key stressors, establish causality and predictive models, and define effect thresholds, that is, PNECs (Chariton et al. 2016). For example, the relative impacts of individual stressors can be expressed using a cumulative stress index in each study site, and then the cumulative stress values of different sites can be jointly analyzed with consideration of these multiple stressors using multivariate analysis (Allan et al. 2013). Based on the results, we can identify the most impacted sites and the corresponding key stressors. Furthermore, the use of ecogenomics (eDNA) approaches can characterize in far more detail the biological community at each site of concern (Yang et al. 2017), and such biodiversity data (number of species, functional groups) can then be treated as a covariate in the analysis. With the availability of big data and advanced statistical analysis tools, this complex question could be adequately addressed in the future. However, the availability of relevant monitoring data may be relatively lacking in many parts of developing Asia, and hence concerted effort in data acquisition would present a challenging and crucial step.

### How can we develop an integrative and effective framework (e.g., environmental policy, green technologies) to manage nutrient loading and associated hypoxia in Asia? (Q14)

Globally, there is an increasing trend in the number of dead zones in the coastal marine environment as a result of eutrophication‐driven hypoxia, with the number of such zones at approximately 500 (United Nations Educational, Scientific and Cultural Organization 2020). The situation in Asia is expected to follow the same trend, though some of the developing countries in the region might not monitor or report hypoxic events. The impact of eutrophication‐driven hypoxia can be catastrophic; a low‐oxygen environment can kill all sessile benthic organisms and have cascading effects on the marine ecosystem, including reduction of fishery resources and an increase of organic matter in sediment that further increases long‐term hypoxia (Breitburg et al. 2018). Furthermore, previous studies suggested that tropical estuaries would be more prone to nitrogen retention via dissimilatory nitrate reduction to ammonia instead of nitrogen loss via denitrification and anammox under anoxic conditions, when compared to temperate systems (Dong et al. 2011; Li et al. 2019). If this is true, this could make marine ecosystems in tropical Asia more prone to eutrophication. Apparently, we need to have a better understanding of nutrient cycling in coastal marine ecosystems in tropical Asia.

To effectively manage such unacceptable situations, an integrated management approach is required to effectively minimize the release of nutrients from agricultural and industrial areas, as well as municipal sewage effluent and surface runoff. A comprehensive framework for controlling nutrient discharge has been suggested by Chen and Hong (2012), which consists of multiple levels of management of human activities and involvement of various stakeholders including intergovernmental cooperation for transboundary management (e.g., if a river is shared between countries).

### How will changes to physicochemical characteristics (e.g., salinization/ion imbalance, pH, temperature, hypoxia due to enrichment) alter the bioavailability and effects of chemical stressors in the environment? (Q15)

This question has some degree of overlap with Question 12, concerning the combined effect of contaminant mixtures and multiple stressors (e.g., pH and temperature) under varying environmental conditions. This is a very complex issue, calling for more well‐thought‐out research studies to enable us to improve our understanding of these compounded effects and make better predictions of chemical toxicity under different climate regimes (e.g., Wang et al. 2016, 2019b; Mu et al. 2018). This question is clearly not restricted to Asia.

It is a truism that rarely do toxicant additions occur in the absence of other changes to water chemistry (e.g., Kienzler et al. 2014; Pan et al. 2015; Bopp et al. 2016; Godoy and Kummrow 2017), yet most knowledge of contaminant ecotoxicity is based on testing of single contaminants without other stressors present. Although there were limited experiments being conducted with a selection of 2 or 3 contaminants or commercial product formulations that are variable, their experimental settings still generally represent relatively simple situations without considering variations in environmental conditions. Consequences of this difference between testing of contaminant toxicity and the reality of environmental exposures can be readily gleaned from careful reading of the general mixture toxicity literature including the reviews cited earlier in this paragraph. Although some attempts have been made in recent years using the multi–biotic ligand model to assess the aquatic toxicity of metal mixtures (Santore and Ryan 2014) and the integrated “simplified” approach for assessing the risk of chemical mixtures (Diamond et al. 2018), this remains a very challenging question to address in the coming decade.

### How can we prioritize and apportion chemical stressors in complex scenarios to guide restoration efforts? (Q16)

Although methods have been developed to assess the overall toxicity of mixtures (sometimes called “whole‐effluent toxicity” tests [Hall and Golding 1998] or “direct toxicity assessment” [Australian and New Zealand Governments 2018], depending on the jurisdiction) and determine the groups of substances contributing to that toxicity (often termed “effects‐directed analysis” or “toxicity identification evaluation” [Brack 2011; Burgess et al. 2013]), these are methods of “hind‐casting” the toxicity of environmental mixtures, not predicting their toxicity. The limited research to date into mixture toxicity means that the ability to predict the toxicity of contaminant mixtures, or to be certain what substances should be prioritized for control, is currently very limited.

The Asia region is particularly prone to existing and emerging contaminant mixtures because of the co‐occurrence of the world's largest national populations, the highest national population growth rates, rapidly developing economies (Table 1), and, commonly, environmental regulation regimes that are developing from a minimal base and/or have limited capacity to enforce compliance. To further complicate the issue, rarely do these mixtures of contaminants enter the environment in the absence of other changes to the physicochemical characteristics of the receiving environment, often being associated with changes in pH, temperature, nutrient levels, or salinity that are associated with the source(s) of the contamination.

Furthermore, the Asia region includes several areas that are particularly sensitive to climate change, including changes to water availability and sea‐level rise (Field and Barros 2014). These factors mean that mixture toxicity impacts are increasingly going to co‐occur with changing receiving environment characteristics. What is urgently needed in the face of these multiple challenges to managing environmental impact to the region are tools to enable proactive prioritization of actions. This will require the ability to predict which of the multiple stressors most urgently needs improved management. Although this is not a problem that is unique to Asia, it is arguably more urgent that these predictive tools and more systematic approaches to proactive environmental management of contaminants be developed for this region with apparent challenges brought by the ever‐growing population, rapid economic development, and urbanization (Hooper et al. 2013).

### How can we identify adverse impacts of multiple stressors in the field to biodiversity (including multigenerational, evolutionary, and developmental), ecosystem services, and human health? (Q17)

Future ecological risk assessments would be based on impacts on ecological function and service, relevant to water and land management and regulatory decision‐making (Maltby et al. 2017, 2018). Ecological function and service often refer to ecological processes (diversities of species and traits) and the resulting properties that support the well‐being of human populations (Gibson et al. 2015). Not only species diversity but also diversity of traits can affect ecosystem function and service. Accordingly, elevated concentrations of chemical mixtures can ultimately cause adverse impacts to the ecosystem services being provided to humans (e.g., provision of clean water and food, enabling healthy cycling of nutrients).

It is, therefore, critical to develop advanced tools that can encompass the biodiversity, community composition (trophic interactions, food web complexity/diversity), and biogeochemical processes that drive ecosystem function in environments of concern (Compson et al. 2018). These tools should support routine application in a consistent and repeatable manner so that they can be easily incorporated into regulatory and management programs (Gibson et al. 2015). For example, eDNA meta‐barcoding can generate DNA sequence–based data for monitoring and predicting the effects of anthropogenic contamination of chemicals on aquatic ecosystems (Li et al. 2018a; see also Question 5).

Mesocosm‐based experiments provide an ideal approach to understand the ecological mechanisms of the effects of different stressors (e.g., pesticides, herbicides, and fertilizers) on biodiversity and ecosystem functioning (Nienstedt et al. 2012). Development of supporting knowledge bases and ecological models (e.g., for food webs) will eventually enhance our capability to differentiate ecological adverse outcomes by different stressors in the field (Halstead et al. 2014).

### *To what extent is seawater pH in Southeast Asia impacted by terrestrial inputs (e.g., organic carbon, nutrients, other anthropogenic sources such as mining), how are these inputs changing as a result of human activities (including CO*_*2*_ *), and how does this affect vulnerable coastal ecosystems such as coral reefs? (Q18)*

In addition to the release of carbon dioxide and reactive nitrogen compounds from the burning of fossil fuel, pH in river, estuary, and coastal water can be greatly influenced by the discharge of various chemical contaminants (Orr et al. 2005). For example, the discharge of mine tailings which are acidic in nature can significantly reduce the pH in receiving water bodies, thereby increasing the bioavailability of metals (Byrne et al. 2012). The reduction of pH may lead to ocean acidification and imbalance of the carbonate system that will negatively affect the growth of corals and other organisms that require calcium carbonate to build their skeleton (Orr et al. 2005). Because there are many mining sites across tropical Asia, the risk of such anthropogenically driven ocean acidification to corals is expected to be high (Burke 2006). More field‐based studies on acidification of estuaries and coastal waters in Asia are warranted to address this important issue and uncover the current status and extent of the impact.

Global food production must increase by 200% to meet the needs of developing countries by 2050. Aquaculture, which surpassed global fisheries for production of fish for human consumption in 2014 (Food and Agriculture Organization 2016), will be critical to meet these needs, particularly for Asia in which per capita seafood consumption is elevated and the majority of global aquaculture production exists. Defining and managing environmental and health challenges in Asia is becoming increasingly important because most of the population already lives in cities, where access to and consumption of chemical products are occurring faster than environmental management systems, making public health interventions necessary (Brooks 2018). In fact, pollution is now responsible for profound health burdens, especially in lower‐ to middle‐income countries (Landrigan et al. 2018). For example, 80% of sewage produced around the world is released untreated to surface waters (World Water Assessment Programme 2017). Unfortunately, terrestrial agriculture and aquaculture efforts are practiced in urban and periurban areas receiving wastewater discharges (Burket et al. 2018). Integrated challenges among urbanization and water and food security are thus pronounced in Asia, where 22 megacities (>10 million people) are projected to exist by 2030 and where over one‐third of the human population will live in 2050 (United Nations 2018).

### How can we develop new technology and promote green chemistry for enhancing reuse of waste and preventing environmental impacts? (Q19)

Sustainable and green chemistry (Anastas and Warner 1998) and engineering (Anastas and Zimmerman 2003) present clear opportunities to meet some of the environmental and health challenges associated with rapid population growth and urbanization in Asia. In fact, sustainable and green chemistry are poised to make significant contributions toward realizing some of the United Nations Sustainable Development Goals (Anastas and Zimmerman 2018; Brooks 2018, 2019). Sustainable molecular design, which is inspired by the fourth principle of green chemistry, specifically aims to design chemicals with lower hazards but that maintain intended functions (Voutchkova et al. 2010; Coish et al. 2016). Such efforts are fuelling innovation, decreasing risks to public health and the environment, and appear important for Asia where robust waste‐management systems remain differentially implemented.

### Given increasing population growth and per capita demand for seafood in Asia, how can we develop sustainable aquaculture practices while protecting environmental quality, particularly in coastal waters? (Q20)

This priority research question identifies a timely need to develop more sustainable aquaculture practices in Asia, which at the same time do not impair environmental quality and are not associated with unacceptable risks for aquaculture product safety (Sapkota et al. 2008; Burket et al. 2018), ecosystem services, and biodiversity (Rico et al. 2012). To address this question, a coordinated effort will be required across the environmental toxicology and chemistry, agriculture, ecology, and public health disciplines. For example, expanding specimen banks that examine contaminants in multiple environmental compartments (soil, sediment, water, food, human tissues) and potential relationships to biodiversity and population health has been identified as a particularly timely opportunity for Asia (Brooks and Conkle 2019).

### How can we develop innovative solid waste‐management programs to protect environmental quality, particularly in rural areas of less developed regions in Asia? (Q21)

Asia is arguably the major contributor of plastic waste to the oceans (Jambeck et al. 2015; Lebreton et al. 2017). With increasing population and rapid economic development in the region, this trend is only likely to worsen. The problem of improper waste management is particularly serious in Asian megacities but also increasingly alarming in rural and newly developing urban areas in this region. Solid waste‐management systems in Asia are overwhelmed because many Asian countries do not have a proper waste‐management system and waste‐treatment infrastructures (e.g., advanced waste‐to‐energy facilities and well‐managed waste‐recycling industries; Jones 2018; World Bank 2018). The decision by China to ban imports of waste for recycling (Lasker et al. 2017; Lee 2018) is an indication of a national system struggling to cope with increasing solid waste production and an example of the far‐reaching implication of waste‐management strategies in one country affecting other countries in the region. However, when it comes to implementing positive changes to waste management, the environmental impact of the current systems is commonly the weakest driver (Agamuthu et al. 2009). Furthermore, this solid waste issue is also closely related to the potential release of complex contaminant mixtures (e.g., release of landfill leachates) because solid waste is regarded as a major source of chemical contaminants. In the face of these critical issues in solid waste management, reliance on continuation of the gradual adaptation of traditional management systems is almost certain to fail. Innovative solid waste‐management systems are, therefore, urgently needed in Asia and many other rapidly growing regions.

### What is the extent of antibiotic pollution in the environment and associated risks of antibiotic resistance in rural and urban regions of Asia? (Q22)

This priority question focuses on understanding the extent to which antibiotic pollution and antibiotic resistance are occurring in the environment, in both urban and rural parts of Asia. This represents an exceptionally important research need because antibiotic resistance is a leading global health threat (World Health Organization 2018). For example, Chung et al. (2018) recently reported multiple antibiotics being discharged by leachate effluents from landfills of Hong Kong that exceeded PNEC thresholds for the development of antibiotic resistance (Bengtsson‐Palme and Larsson 2016). Antibiotics exceeding PNECs from closed landfills and one of the largest active landfills in Asia included erythromycin and ciprofloxacin (Chung et al. 2018). Both erythromycin and ciprofloxacin are designated as critical antibiotics by the World Health Organization (2011). Such observations require additional study in Asia because erythromycin exceedances in effluents of Asia are higher than in Europe and North America (Schafhauser et al. 2018), while reported global effluent discharges of ciprofloxacin exceed antibiotic resistance development thresholds 58% of the time (Kelly and Brooks 2018). It is also important to note that similar research questions were identified during GHSP efforts in Oceania (Gaw et al. 2019), North America (Fairbrother et al. 2019), and Latin America (Furley et al. 2018), which face similar challenges to the developing regions in Asia.

### How can we develop sustainable development frameworks (e.g., green chemistry) to address, balance, and manage the production (e.g., food production, forestry) and protection of ecosystem services? (Q23)

This priority question is closely related to Question 19 that targets the need to develop innovative technologies to advance waste resource recovery while improving environmental quality. Question 23 specifically identifies the research niche to derive integrative sustainable development frameworks that incorporate science and technology advances from green chemistry and engineering to balance agricultural and natural resource production while protecting ecosystem services. Related priority questions were also identified during the GHSP efforts in the Australasian region of Oceania (Gaw et al. 2019), Europe (Van den Brink et al. 2018), and North America (Fairbrother et al. 2019), which are directly relevant to other continents (e.g., Africa, Latin America).

## OUTLOOK

The 23 priority research questions identified here for Asia exemplify the diversity and environmental and human health challenges facing the region. Whereas many of these questions engage cutting‐edge topics, others identify research needs that are particularly germane to low‐ and middle‐income countries in Asia and around the world. Recent HSP exercises have been conducted in Europe (Van den Brink et al. 2018), Latin America (Furley et al. 2018), North America (Fairbrother et al. 2019), and the Australasian region of Oceania (Gaw et al. 2019). As noted in Table 2, priority questions vary among regions, though some common topics are emerging. Several research questions are uniquely identified from Asia, including Questions 3, 11, and 18 (Table 2), which deal with issues related to air pollution, application of nanotechnology to advance ecotoxicology, and impacts of carbon dioxide and ocean acidification. It is important to note that several Asian questions (e.g., Questions 13, 19, and 23) are common with other regions and thus appear globally important (Table 2). For instance, Question 13, addressing the combined influences of changing landscapes, climate change, and chemical pollution on ecosystem responses, is a universal priority question across all regions and reflects several global megatrends. Moreover, the other common questions cover 1) development of high‐throughput screening tools for advancing risk assessment of chemicals; 2) identification of adverse impacts of multiple stressors to biodiversity, ecosystem services, and human health; and 3) establishment of sustainable development frameworks, including sustainable and green chemistry and engineering. Question 22, which is related to antibiotic resistance, was identified in several other regions, and particularly highlighted in Latin America, perhaps because of similarities in waste‐management capacity with the low‐ and middle‐income countries in Asia.

The Asia Pacific region needs solutions to these pervasive problems, and it needs them now. Although many of these issues have strong socioeconomic and political drivers, environmental quality in the receiving ecosystems of the region urgently needs better prediction and management tools. Given that these problems are routinely pronounced for rapidly developing economies in Asia with the most limited economic, regulatory, and social capacity to deal with them, these tools need to be accessible and efficient to have the level of impact needed. This set of 23 questions will be very challenging to address, but it is critically important that they are. This research effort is deemed to be necessary for improving environmental quality and promoting sustainable development in Asia. Future efforts supporting country‐specific and international activities, including nontraditional partnerships, are needed to engage the priority research questions identified here.

## Data Availability

Data, associated metadata, and calculation tools are available from the corresponding author (kmyleung@hku.hk).
